# Intrinsically disordered regions in TRPV2 mediate protein-protein interactions

**DOI:** 10.1038/s42003-023-05343-7

**Published:** 2023-09-22

**Authors:** Raghavendar R. Sanganna Gari, Grigory Tagiltsev, Ruth A. Pumroy, Yining Jiang, Martin Blackledge, Vera Y. Moiseenkova-Bell, Simon Scheuring

**Affiliations:** 1 Department of Anesthesiology, Weill Cornell Medicine, 1300 York Avenue, New York, NY 10065 USA; 2 Department of Systems Pharmacology and Translational Therapeutics, Perelman School of Medicine, University of Pennsylvania, Philadelphia, PA 19104 USA; 3 Institute of Structural Biology, Perelman School of Medicine, University of Pennsylvania, Philadelphia, PA 19104 USA; 4 Biochemistry & Structural Biology, Cell & Developmental Biology, and Molecular Biology (BCMB) Program, Weill Cornell Graduate School of Biomedical Sciences, New York, USA; 5 Université Grenoble Alpes, Centre National de la Recherche Scientifique (CNRS), Commissariat à l’Énergie Atomique et aux Énergies Alternatives (CEA), Institut de Biologie Structurale (IBS), 38000 Grenoble, France; 6 Department of Physiology and Biophysics, Weill Cornell Medicine, 1300 York Avenue, New York, NY 10065 USA

**Keywords:** Atomic force microscopy, Ion transport

## Abstract

Transient receptor potential (TRP) ion channels are gated by diverse intra- and extracellular stimuli leading to cation inflow (Na^+^, Ca^2+^) regulating many cellular processes and initiating organismic somatosensation. Structures of most TRP channels have been solved. However, structural and sequence analysis showed that ~30% of the TRP channel sequences, mainly the N- and C-termini, are intrinsically disordered regions (IDRs). Unfortunately, very little is known about IDR ‘structure’, dynamics and function, though it has been shown that they are essential for native channel function. Here, we imaged TRPV2 channels in membranes using high-speed atomic force microscopy (HS-AFM). The dynamic single molecule imaging capability of HS-AFM allowed us to visualize IDRs and revealed that N-terminal IDRs were involved in intermolecular interactions. Our work provides evidence about the ‘structure’ of the TRPV2 IDRs, and that the IDRs may mediate protein-protein interactions.

## Abstract (web summary)

High-speed AFM enables visualization of intrinsically disordered regions (IDRs) in TRP ion channels and shows that IDRs may mediate protein-protein interactions with adjacent TRP molecules.

## Introduction

Transient receptor potential (TRP) channels are the second largest class of ion channels comprising six subfamilies: TRPV (vanilloid), TRPM (melastatin), TRPC (canonical), TRPA (ankyrin), TRPP (polycystin) and TRPML (mucolipin)^1^. These channels are activated by diverse stimuli including chemical and physical stimuli^2^. TRP channels play important roles in cellular processes, such as sensory and signal transduction, and their malfunction is associated with polycystic kidney diseases, respiratory diseases, and several types of cancers^3^. Because of their role in pathophysiology, many TRP channels are considered as potential drug targets.

All TRP channels are tetramers where the ion conducting pathway, the pore, is located at the 4-fold symmetry axis - though we recently discovered that TRPV3 could adopt transiently and reversibly a pentameric state^4^. The recent improvements in cryo-EM and image analysis enabled the resolution of almost all TRP channel structures, i.e., at least several members of each subfamily^1,5^. All available structures show that the individual TRP channel subunit consists of six transmembrane α-helices (S1-S6), a cytoplasmic N-terminal domain with a variable number of ankyrin repeats, and a C-terminal region. It is important to note that most structures do not resolve the N- and C-terminal regions, likely due to their inherent flexibility. Indeed, a recent analysis suggested that TRP channel N- and C-termini comprise extended stretches that are intrinsically disordered regions (IDRs), which can constitute 25 to 50% of the total sequence in some TRP channels^6^.

The TRPV (vanilloid) subfamily consists of six channels grouped into two categories: heat-activated non-selective Ca^2+^-channels (TRPV1-V4) and ion selective non-thermal Ca^2+^-channels (TRPV5 and TRPV6)^1,5^. Several structures of all six TRPV channels have been solved^1,5^. The large intracellular N- and C-terminal regions of TRPV channels contain putative protein interaction- and regulatory motifs. For instance, the ankyrin repeat domain (ARD) located in the N-terminal region has been suggested to play a role in phosphorylation and binding of regulatory proteins^7,8^. ARDs have also been reported to be implicated in protein-protein interactions^9^. In TRPV4, phosphatidylinositol-4,5-biphosphate (PIP_2_) was shown to interact with and rearrange the N-terminus facilitating channel activation^10^. All TRPV1-4 structures lack densities for the N- and C-terminal IDRs, corroborating the high flexibility of these protein moieties. Recent structural work on TRPV2 and TRPV3 resolved portions of the N- and C-terminal regions, suggesting that at least some stretches of the N- and C-terminal IDRs may adopt conserved structure under specific conditions^11–13^. While the IDRs are being recognized as important contributors to the TRPV channels activity, their characterization is sparse. Previously, Förster or bioluminescence resonance energy transfer (FRET and BRET) assays have been used to study the arrangements of N- and C-terminal tails of TRPV channels in cells, but resolution limitations precluded to resolve these flexible parts^14,15^. Thus, the structure and dynamics of IDRs remain experimentally unexplored, and the elucidation of the whereabouts of these regions and their potential interplay in the formation of protein interactions remain a missing piece in our understanding of TRPV proteins.

Over the last decade, high-speed atomic force microscopy (HS-AFM) has provided crucial molecular insights into many biomolecules including DNA, soluble proteins, membrane associate proteins, and transmembrane transporters and channels^16–19^. HS-AFM imaging reaches nanometer lateral, Angstrom vertical and sub-second temporal resolution in aqueous environment and at ambient temperature and pressure. Recently, we introduced a super-resolution method for AFM data that allowed to break the tip-convolution restricted nanometer lateral resolution limitation and permitted to extract Angstrom resolution structural details on membrane protein surfaces^20^. HS-AFM has the additional advantage that the environmental cues, such as the buffer composition^21^, light, temperature^22^, and force^23^, can be altered during movie acquisition.

It is predicted that ~40% of all eukaryotic proteins consist of and/or contain intrinsically disordered regions (IDRs) that do not adopt well-defined secondary or tertiary structure^24^. IDRs are known to play an important role in protein-protein interactions, post-translational modifications, and degradation by proteasomes^24^. Despite their abundance and functional significance, structural and dynamic information about IDRs has been challenging to obtain experimentally. NMR and SAXS have been used for the structural analysis of IDRs^25,26^, however, ensemble averaging by these techniques put limitations to the extractable information. Single molecule FRET is capable to analyze the structure and dynamics of IDRs^27,28^, but is restricted to distance measurements only and within length limitations <10 nm. Recently, owing to its high signal-to-noise ratio on the single molecule level, HS-AFM has proven powerful to investigate structure and dynamics of intrinsically disordered proteins (IDPs)^29,30^. These studies indicated the potential use of HS-AFM to analyze IDRs in the context of membrane proteins.

Here, we performed HS-AFM imaging of membrane-reconstituted full-length TRPV2 channels. Our results show that the TRPV2 N- and C-terminal regions form highly flexible IDRs, which can however be probed by HS-AFM and structurally characterized. Furthermore, we show that the IDRs engage in protein-protein interactions with adjacent TRPV2 molecules.

## Results

### TRPV2 membrane reconstitution for HS-AFM analysis

**Fig. 1:**
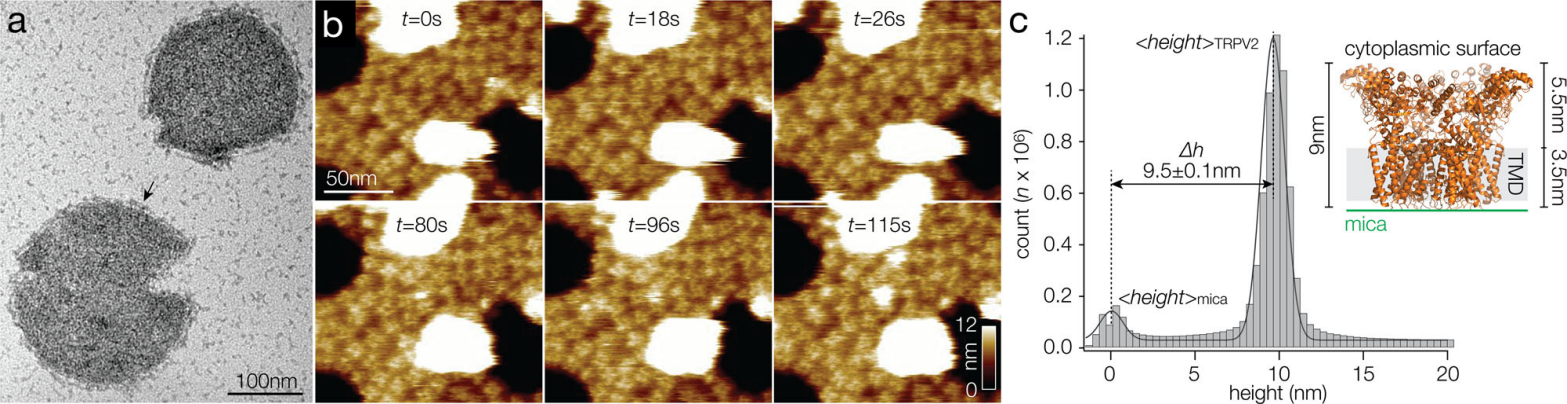
TRPV2 reconstitution for HS-AFM analysis. **a** Negative-stain EM of TRPV2 reconstituted into soy polar lipids at a lipid-to-protein ratio of 0.7. Protruding features (arrow) at the vesicle periphery and the strong contrast of the proteins in the vesicle in the negative-stain EM are indicative of inside-out reconstitution of the TRPV2 channels with the large cytoplasmic domains exposed to the outside of the vesicle. **b** Overview HS-AFM images (Supplementary Movie 1) of TRPV2 (windmill-shaped molecules) in soy polar lipid membranes on mica (dark background areas). False color scale: 0–9 nm. The white oversaturated areas have a height of ~26 nm and represent likely non-ruptured small vesicles. **c** Height distribution of TRPV2 above mica from (**b**). TRPV2 has a full height of 9.5 ± 0.1 nm above mica, in good agreement with the TRPV2 cryo-EM structure. Inset: Cryo-EM structure PDB 6U84 shown with the intracellular side up (as imaged by HS-AFM), membrane indicated in light gray.

We reconstituted purified full-length TRPV2 into soy polar lipids at lipid-to-protein ratios (LPR) between 0.5 and 0.7 (see “Methods”). Negative-stain electron microscopy (EM) showed that reconstitution resulted in proteo-liposomes of up to ~200 nm in size that were densely packed with TRPV2 molecules (Fig. 1a). The membrane protruding structures at the brim and the strong staining of the flattened areas of the proteo-liposomes, where the stain visibly can surround the strongly protruding intracellular surface of the TRPV2 channels resulting in high contrast, was evidence for inside-out packing of the channels in the proteo-liposomes. The proteo-liposomes were deposited on freshly cleaved mica and imaged by HS-AFM in buffer solution (see “Methods”). HS-AFM allowed to record time-lapse movies at 1 to 6 frames per second scan rate over minutes (Fig. 1b and Supplementary Movie 1). Height histogram analysis reported that TRPV2 had a height of 9.5 ± 0.1 nm above the mica background (Fig. 1c), in excellent agreement with the overall height of ~9 nm of the TRPV2 cryo-EM structure^31–33^, where the transmembrane domain (TMD) spans ~3.5 nm and the cytoplasmic domain protrudes ~5.5 nm from the membrane (Fig. 1c, inset). We attribute the minor difference in height 9.5 nm (HS-AFM) vs. ~9 nm (cryo-EM structure) to the short extracellular loops and/or a thin buffer layer between the protein packed membrane and the mica surface. Since the extracellular face of TRPV2 protrudes only faintly above the membrane and only constitutes of small loops, all our measurements focus on the intracellular side where the strongly protruding windmill-shaped ARDs and the IDRs are accessible to the HS-AFM probe.

### Intrinsically disordered regions (IDRs) at the TRPV2 perimeter

**Fig. 2:**
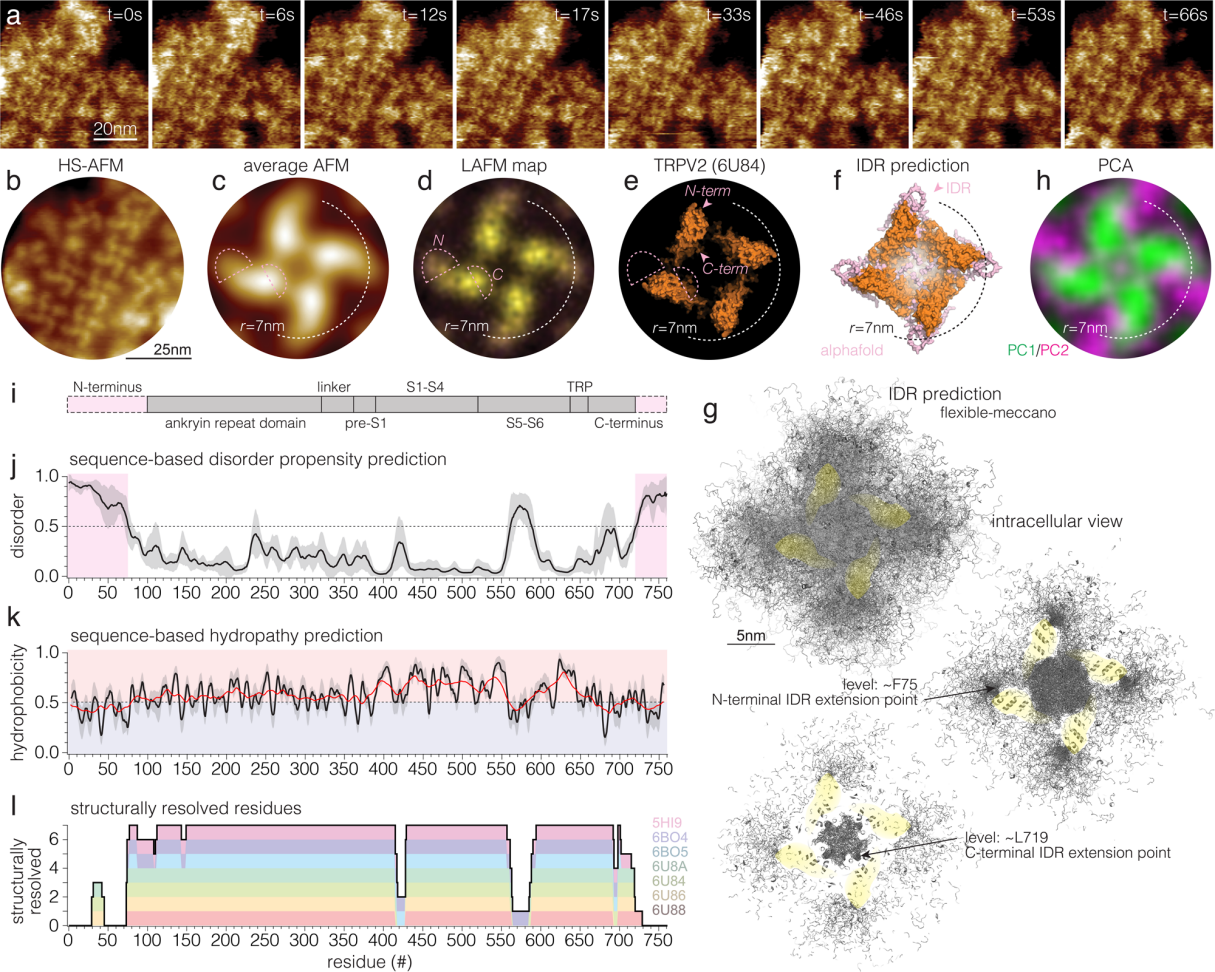
The “structure” of intrinsically disordered regions (IDRs) in TRPV2. **a** High-resolution HS-AFM images of TRPV2 channels (Supplementary Movie 2). **b** HS-AFM image (time average over 5 frames from (**a**)) of TRPV2 channels with markedly elongated and left-handed twisted protomer structure. **c** Average AFM map (*n* = 532 particles). **d** Localization AFM (LAFM) map (*n* = 532 particles, *n* = 80,000 localization detections). **e** Surface representation of TRPV2 cryo-EM structure (6U84). **f** TRPV2 AlphaFold structure prediction shown in surface representation: the core structure (orange) is extended by the predictions of the N- and C-terminal IDRs (pink) that do not comprise secondary structures. The structures of the termini are predicted as with low confidence to likely unstructured (see Fig. S1). **g** Flexible-meccano IDR structure prediction. 5000 structures for each subunits’ IDRs (20,000 N- and 20,000 C-termini) are shown as gray lines. Top: View on the cytoplasmic face. Middle: 12 Å slab around the position of F75, where the N-terminal IDRs emerge from the molecular periphery. Bottom: 12 Å slab around the position of L719, where the C-terminal IDRs emerge toward the central cavity. **h** Principal component analysis (PCA) of TRPV2 molecules with PC1 (green, most shared features) and PC2 (magenta, second most shared features, orthogonal to PC1). Dashed hemi-circle in (**c**–**g**) outline the peripheral limits of the cryo-EM structure at *r* = 7 nm. Pink dashed outlines in (**c**–**e**) highlight the area where HS-AFM detects additional protein structure assigned to the N- and C-terminal IDRs, absent in the cryo-EM structure. Arrowheads in (**e**) indicate the locations of the first and last commonly resolved residues, F75 and L719. **i** Linear diagram of the TRPV2 sequence with unresolved N- and C- termini depicted in pink and all other domains depicted in gray. **j** Disorder score based on sequence (using PONDR VLXT, PONDR VL3, PONDR VLS2, IUPRED2, PONDRFIT, PRODOS; see “Methods”). Line and shaded area: average ± standard deviation of the predictions from all software. **k** Hydrophobicity score based on sequence hydropathy prediction (using ExPasy-protScale; see “Methods”). Line and shaded area: average ± standard deviation of the predictions from various hydropathy scales (see “Methods”). Red (>0.5) and blue (<0.5) shaded areas depict more hydrophobic or hydrophilic scores, respectively. The red trace is a smoothened trendline, a walking-average over 20 consecutive residues illustrating the hydrophilic trends on both the N- and C-terminal regions. **l** Structurally resolved residues in the seven currently available *Rattus norvegicus* TRPV2 structures (independent of conditions). The disorder propensity (**j**) and structurally resolved regions (**l**) correlate.

Next, we acquired high-resolution HS-AFM images of TRPV2 membranes to watch individual channels over time at high spatio-temporal resolution (Fig. 2a and Supplementary Movie 2). TRPV2 molecules in HS-AFM images featured extended intracellular domains with a noteworthy left-handed twist (Fig. 2a, b). Calculation of a correlation average map corroborated the overall windmill-shaped molecular architecture that has been observed on the single molecule level (Fig. 2c), while merging local protrusion maxima in a localization AFM (LAFM^20^) map revealed additionally sub-molecular details (Fig. 2d) that corresponded well to the surface features of the ARD repeats in the molecular structure (Fig. 2e). However, the averaging processes to calculate these maps obliterated to some degree the full extent of the flexible and bent TRPV2 wings, as they arguably spread longer in individual protomers of the raw data (Fig. 2a, b). Regardless, comparison of these maps (Fig. 2c, d) and of the individual topographs (Fig. 2b) with the surface of the cryo-EM structure (Fig. 2e) indicated that HS-AFM tracked more of the protein surface than is currently resolved by cryo-EM: In contrast to the current cryo-EM structure that has a maximum radius of 7 nm (as measured from the four-fold axis to Cα of Trp101, Fig. 2e), HS-AFM revealed additional protein topography closer to the four-fold axis and depicted the intracellular domains of TRPV2 to reach further beyond the base of the neighboring subunit with a ~20° left-handed twist (Fig. 2b–d). What could these additional protein moieties be?

To get further insights into the “structure” of these moieties, we used AlphaFold^34^ to predict the full-length TRPV2 structure, focusing on the predictions of the protein stretches that remained unresolved in the cryo-EM structures (Fig. 2f, pink): the algorithm suggested that the termini comprised no secondary structure and were likely disordered (Supplementary Fig. 1). However, the prediction placed, the N-terminus as a loop-like extension beyond the ~7 nm radius of the cryo-EM structure with a slight left-handed bend. The location and “structure” of the AlphaFold N-terminus is likely driven by the fact that some of the cryo-EM structures resolve a short N-terminal stretch, residues ~30 to ~45 (see Fig. 2l, left), on the membrane facing side of the ARDs. The C-terminus was predicted as an extension toward the channel center (Fig. 2f, pink). While we consider AlphaFold only as a predictive tool, these predictions fit the additional topography recorded in the elongated left-handed HS-AFM structure (Fig. 2b–d, pink dashed outlines in (c) and (d)). Alternatively, we used flexible-meccano^35^ to generate an ensemble of thousands of possible “structures” of the N- and C-terminal IDRs (Fig. 2g). Flexible-meccano was specifically developed to create structural ensembles of disordered peptides using amino acid-specific conformational potentials and volume exclusion to be compared with experimental data^36^. Showing an overlay of 20,000 IDR models (5,000 per subunit) viewed from the cytoplasmic face provides a visual representation of the channel as a fuzzy cross where the channel structure essentially disappears behind the many possible IDR termini configurations (Fig. 2g, top). Visualizing a slab at the approximate height level of the F75, from where the N-terminal IDRs extend, illustrates as high-density regions the most likely continuation of the N-terminal stretches (Fig. 2g, middle) and corroborates the left-handed additional topography found in HS-AFM data (Fig. 2c, d). Visualizing a slab at the approximate height level of the L719 from where the C-termini extend, shows how the tetramer’s central cavity is filled with these IDRs (Fig. 2g, bottom), with high-density regions where the AFM correlation average and LAFM maps revealed additional topography (Fig. 2c, d). Alternatively, the flexible-meccano structural ensemble can be symmetrized and visualized as a 3D density cloud around the channel, illustrating the extent, >5 nm, of the IDRs (Supplementary Fig. 2). Thus, while AlphaFold^34^ proposes a single most likely (yet low confidence) structure of the termini (Fig. 2f) taking advantage of the known orientation and position of the ~15 residues that were resolved on the membrane facing side of the ARDs at least under some conditions in some structures, flexible-meccano^35^ gives us an image of the structural ensemble of lengths and orientations that free IDR termini could adopt around the channel. Both computational methods agree with the HS-AFM data that the N-terminal IDR extensions prolong the ARDs with a slight left-handed kink, AlphaFold^34^ with a low-confidence loop, and flexible-meccano^35^ with a high positional probability.

Based on these analyses, we suggest that our HS-AFM data captured the average “structure” of the intrinsically disordered termini. To further corroborate the identification of such regions in our data, we used principal component analysis (PCA): In PCA a covariance matrix is calculated between the identical pixels of the images of particles of a time-lapse recording, and the eigenvectors characterize the principal components (PCs) of the data, where PC1 represents the most shared features between all particles and PC2 the second most shared features that are fully independent from the features of PC1 (PC2 lies orthogonal to PC1). Thus, principal components 1 (Fig. 2h, PC1, green) and 2 (Fig. 2h, PC2, magenta) have no overlay, and PC1 represents the channel core much alike the features that are enhanced and preserved through averaging (Fig. 2c) and conserved in the cryo-EM structure (Fig. 2e), while PC2 comprises the components that are variable. Notably, PC2 highlights features at the windmill-like protomer peripheries and in between protomers (Fig. 2h). Thus, the topography in PC2 at the periphery of the intracellular domains coincide with the locations of the N-terminal IDRs and extend them even further, providing direct experimental evidence for such regions at the molecular level and their flexible nature separating them from PC1.

Inspired by a recent report^6^ in which the missing regions in TRP channel structures were compared to the location and abundance of IDRs in the amino acid sequence (Fig. 2i), we calculated the disorder propensity using several IDR sequence predictions algorithms (Fig. 2j, see “Methods”). Accordingly, disorder propensity scores above 0.5 correspond to an unstructured region, indicating that the first ~75 residues, a stretch in the S5-S6 pore region, and the last ~40 residues in TRPV2 were disordered (Fig. 2j). Further, sequence-based hydropathy prediction showed that these IDRs are hydrophilic in nature, where the first ~75 and the last ~40 residues are among the most hydrophilic (Fig. 2k, black line), highlighted through averaging over stretches of 20 residues along the sequence (Fig. 2k, red line). This analysis clearly shows that these termini are not membrane-embedded or -interacting but rather extend into the bulk or interact with bulk exposed parts of the channel. Finally, these sequence-based predictions (Fig. 2j, k) match quite astonishingly well the unresolved regions in the available *Rattus norvegicus* TRPV2 cryo-EM structures (Fig. 2l): Indeed, the TRPV2 cryo-EM structures miss densities for the N-terminal ~75 and the C-terminal ~40 amino acid residues. This corresponds to ~8.3 kDa N-terminal and ~4.4 kDa C-terminal missing protein moieties per protomer. Next, we inspected, where the first, F75, and the last, L719, commonly resolved residues were located in the TRPV2 cryo-EM structure and saw that the missing N-termini should be located at the periphery of the ARDs and the missing C-termini toward the channel four-fold axis (Fig. 2e, arrowheads). Thus, a total of ~33 kDa polypeptide should surround each tetramer and an additional ~18 kDa polypeptide should locate in the intracellular cavity surrounded by the ADRs. In addition, the cryo-EM structure served us a control of the structure in absence of the IDR termini. Altogether, these analyses provided evidence that the N- and C-termini formed extended, hydrophilic IDR stretches that precluded their resolution in cryo-EM. HS-AFM raw data, cross-correlation averaging, and LAFM suggested that the IDRs extended as ~20° bent left-handed extensions from the ARDs (N-termini) and toward the 4-fold axis (C-termini). PCA of the HS-AFM data, AlphaFold and flexible-meccano modeling support these findings.

### IDR mediated protein-protein interactions

**Fig. 3:**
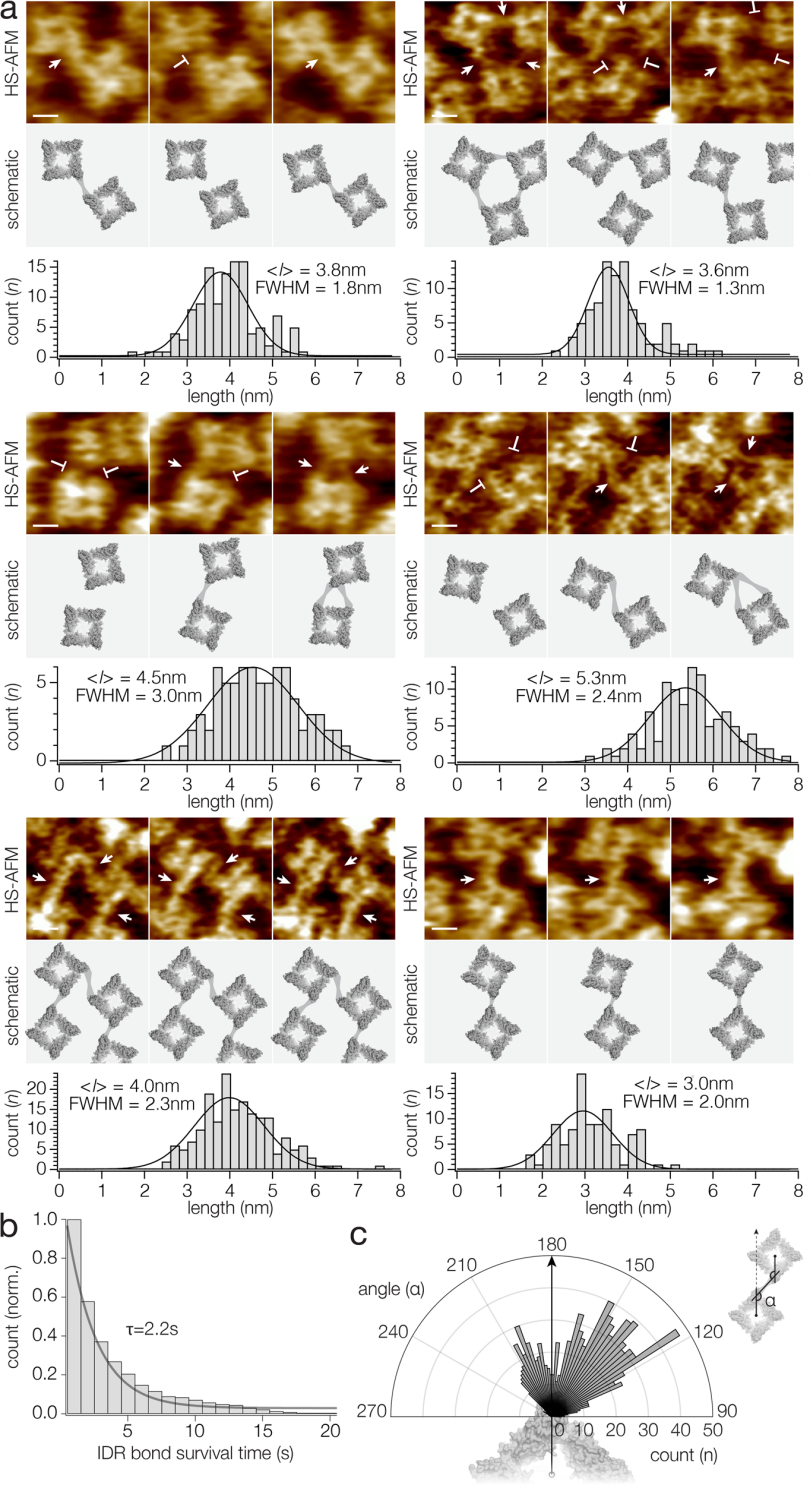
IDR-mediated TRPV2-TRPV2 interactions. **a** Top panels: representative HS-AFM frames of TRPV2-TRPV2 interactions (Supplementary Movies 3–6). Sites between molecules where IDRs interact are indicated by arrows, while the same sites are indicated by blunt lines where the same IDR connections are disengaged. Bottom panels: schematics with connections indicated by lines. **b** Survival plot of protein-protein interactions. Single exponential fitting to the distribution resulted in connection lifetime time constants of 2.2 s (thick contiguous connections with durations exceeding the observation time windows were excluded from this analysis). **c** Polar plot of the angular distribution of IDR interactions. The graph is underlaid with the surface representation of the cryo-EM structure for intuitive illustration how the angular plot relates to the protein orientation. Inset: Diagram illustrating the definition of the measured IDR connection angle as the counter-clockwise enclosing angle between the two lines: (i) tetramer center-to-subunit engaging in connection and (ii) subunit engaging in connection-to-neighboring subunit engaging in connection.

TRPV channel ARDs have been implicated in protein-protein interactions^37^. From our HS-AFM time-lapse imaging we observed that flexible IDRs extended the range of the ARDs (Fig. 2). Using sequence analysis and estimating the reach of the IDRs using a worm-like chain polymer extension model, it has been calculated that each TRPV2 protomer could increase its radius of interaction >5 nm by means of the IDRs, were they to extend from the protein core as freely floating polymer chains^6^. This theoretical assessment is reflected by the flexible-meccano IDR modeling without constraints that illustrates how the N-terminal IDRs reach in some configurations >5 nm beyond the protein core (Fig. 2g). Alternatively, due to the interaction of a stretch (residues ~30 to ~45) in the N-terminal IDRs with the membrane facing side of the ARD, as reported in some cryo-EM structures (Fig. 2l, left), the extent of the IDRs could be shorter (Figs. 2f and S1). Likely the IDRs experience both unconstrained exploration of the accessible space and sporadic interactions with the protein core from which they emerge. Additionally, the IDRs might engage in other preferential interactions if other molecules to interact with were in the vicinity. Thus, we set out to investigate whether we could provide direct experimental evidence for long-range TRPV2 interactions with neighboring molecules. We found plethora of TRPV2-TRPV2 interactions, where IDRs from two neighboring channels mediated inter-protomer contacts in a wide variety of angular configurations, corroborating the extremely flexible nature of the IDRs (Fig. 3a, top and Supplementary Movies 3–6). In some channel pairs, the intermolecular bridges were quasi-indistinguishable from the rest of the intracellular domains, and it was unclear where one molecule ended and the next began (Fig. 3a, last two panels). These thicker intermolecular bridges were stable over extended observation periods. In other channel pairs, the connections were thin and intermittent (Fig. 3a, first four panels). The differences in thickness and lifetime of the intermolecular connections are suggestive of an interpretation where the thicker connections correspond to an intertwining of IDRs from both contacting protomers, while the thin ones are suggestive of an individual IDR extension. We also found examples where an individual subunit formed two thin connections to two neighbor protomers, and thus at least one of these two connections had to be a single receiving IDR connection (Fig. 3a, middle two panels). The lengths of these interactions beyond the cores of the two interacting subunits depended strongly on the center-to-center distance and respective orientation between the two interacting channel tetramers and ranged from 3.0 to 5.2 nm (mean values), where each IDR connection had a broad length distribution with a width of ~2 nm (full width at half maximum, FWHM) (Fig. 3a, bottom). The length variability between connections and within connections over time appears to be a signature of the flexibility of such IDR connections. Modeling of the IDR provides a visual intuition how such disordered termini can form connections over such large distances (Fig. 2g). To estimate the lifetimes of these interactions we plotted their survival time, and exponential fitting resulted in a time constant of ~2.2 s (Fig. 3b). In this survival plot the thick apparently continuous intermolecular connections were excluded, as these connections exceeded the HS-AFM observation time window. Finally, we assessed the angular distribution of IDR contacts to estimate how IDRs mediate TRPV2-TRPV2 intermolecular connections (Fig. 3c). To this end we measured the counter-clockwise enclosing angle, α, between two lines, the first is the line between the 4-fold axis and the subunit that engages into the IDR connection, and the second is the line between this subunit and the neighboring subunit with which the IDR connection is engaged (Fig. 3c, inset): In this setting a straight radial extension of the tetramer center-to-subunit line corresponds to an angle of 180°. This analysis revealed that IDRs explore angles in the range between 90° and 240°. However, the plot showed a bimodal distribution of angles, with most connections in the 120° to 150° range, and a lesser populated interaction around 210°. The 120°–150° connections roughly represent a prolongation of the ARD domain with a slight left-handed twist (Fig. 3c), and we therefore suggest that these connections are N-terminal IDR extensions (see Fig. 2b–h). On the other hand, the population of connections around 210° might represent receiving IDR connections. Therefore, based on the IDR connection angle polar coordinate plot (Fig. 3c), we propose that while the IDRs have a wide range of flexibility, they may not be entirely free to bond at any angle. There are a variety of factors that could contribute to this preferential orientation, including steric limitations where a receiving TRPV2 can be located, and the range of connectivity may be different with other partner proteins. Based on the observation of very long-lived and thick IDR connections (Fig. 3a, bottom) we consider that IDRs might be able adopt some folded state when they engage into specifically favorable interactions with a neighboring IDR. TRPV2’s ability to engage into such physical interactions leads us further to hypothesize that IDRs may have important roles in organizing TRPV channels in a cellular setting (see “Discussion”).

## Discussion

At the time this manuscript is written, more than 210 near full-length (>450 residues) structures have been solved from more than 20 TRP channel families^6^. For the TRPV channel subfamily, several structures of all TRPV1-6 members have been solved. However, all these structures are incomplete, lacking density for protein stretches, mainly in the N- and C-terminal regions^6,38^. Related to this, sequence analysis and NMR chemical shift assignments indicated that TRP channels feature extended regions in their N- and C-termini that are IDRs^6,38^. However, while it has been shown that these regions are important for channel function and regulation^13^, little is known about TRP channel IDRs.

Here, we used HS-AFM imaging to study membrane-embedded TRPV2 at the single molecule level (Fig. 1). Importantly, we found topographic signals that extended the ARDs on the intracellular side. Using LAFM for the extraction of structural features from single molecules, we describe the TRPV2 IDRs to extend the intracellular ARDs preferentially in a left-handed windmill architecture with a ~20° bend and ~2 nm length. While PCA segregated the structurally stable features from the fluctuating parts at the periphery of the channel, sequence analysis, AlphaFold and flexible-meccano predictions provided further evidence assigning the newly detected peripheral protein moieties to the missing IDRs (Fig. 2).

Further, HS-AFM single molecule imaging provided direct visual evidence of the formation of IDR molecular extensions from and intermolecular connections between TRPV2 channels (Fig. 3a). Goretzki et al.^6^ proposed to estimate the length that IDRs can reach using the worm-like chain model^39,40^. We have reproduced these calculations for TRPV2 and found that the average reach of the IDRs was ~4.6 nm beyond the currently resolved channel structure. It is notable that the ARDs extend into the disordered N-terminus at the very periphery of the currently resolved TRPV2 structure, thus the IDRs add substantial interaction range to the channel. This is well reflected by the flexible-meccano ensemble IDR prediction, though performed in the absence of a membrane, that shows the N-termini extending ~8 nm beyond the channel core structure (Fig. 2g), with a dense extension population to >5 nm (Supplementary Fig. 2). Experimentally, we determined that the IDRs could form protein-protein contacts with lengths ranging up to ~5 nm, lifetimes in the seconds time range, and variable angular orientations (Fig. 3b). We found that IDR connections had somewhat preferential orientation around a prolongation of the ARDs from where the N-terminus extends, though this might be constrained in the case of TRPV2-TRPV2 interactions. We further found IDR connections between protomers on the distal protomer face, which could represent receiving IDRs (Fig. 3c). While we operate our HS-AFM with optimized feedback operation minimizing the impact on the sample, we cannot rule out that the HS-AFM tip-sample interaction influences the IDR distributions and interactions. Considering an energy contribution by the oscillating tip during the imaging of the IDRs, we would expect that the lifetime estimate that we provide here is a lower bound, and IDR-mediated protein-protein interactions might be more stable than reported here.

The ability of HS-AFM to image individual TRP channel molecules allowed us here to characterize the structure of unstructured parts of a TRP channel and reveal that these IDRs form stable connections between channels over seconds. We hypothesize that the physical interaction of the AFM tip with the protein enables this performance, despite the IDR flexibility: Likely, the flexible extensions probed by electrons in cryo-EM studies are lost in the averaging process. In contrast, the AFM tip just “cannot not probe” the ~8.3 kDa N-terminal and ~4.4 kDa C-terminal protein moieties, thus giving topographic signal in every single molecule. Given the abundance of TRP channels, their importance in physiological processes and the conservation of IDRs in their N- and C-terminal regions, we anticipate that IDRs represent versatile sites for protein-protein interactions, which may play a role in the organization of TRPV2 and other TRP channels in the membrane.

## Methods

### TRPV2 expression and purification

Rat TRPV2 with C-terminally located 1D4-tag was overexpressed in *Saccharomyces cerevisiae* cells (BJ5457 *S. cerevisiae* (ATCC)). TRPV2 containing membranes were extracted and incubated with solubilization buffer containing 20 mM Hepes, pH 8.0, 150 mM NaCl, 0.87 mM lauryl MNG (LMNG, Anatrace), 5% glycerol, 1.0 mM dithiothreitol (DTT) and 1.0 mM phenylmethylsulfonyl-fluoride for 1 h at 4 °C. After spinning down insolubilized membrane fractions by ultracentrifugation at 100,000 × *g*, 4 °C for 45 min, solubilized fractions were incubated overnight with cyanogen bromide-activated-Sepharose 4B beads (GE Healthcare) attached with 1D4 antibody at 4 °C. TRPV2 was affinity purified by washing with buffer containing 20 mM Hepes, pH 8.0, 150 mM NaCl, 0.064 mM decyl-MNG (DMNG, Anatrace), and 1.0 mM DTT and eluted with 3.0 mg ml^−1^ of 1D4 peptide in the washing buffer. TRPV2 was further purified by size-exclusion chromatography utilizing Superose 6 Column (GE Healthcare). Finally, purified TRPV2 fractions were collected and concentrated with 100 kDa molecular weight cut-off concentrators (GE Healthcare) to ∼1 mg/ml.

### TRPV2 reconstitution

Purified TRPV2 was reconstituted into soybean polar lipid (PC: 45.7%, PE: 22.1%, PI: 18.4%, PA: 6.9%, unknown: 6.9%; Avanti polar lipids) at lipid-to-protein ratios (LPR) between 0.5 and 0.7. Briefly, a lipid film was formed in a glass tube by drying lipids in chloroform using argon gas and by leaving overnight in the desiccator. The lipid film was hydrated with milliQ water and bath sonicated for 30 min to make lipid vesicles. Then vesicles were solubilized with DMNG. Desired quantities of solubilized lipids and protein (for LPR 0.5 and 0.7) were mixed for 6 h at 4 °C. Detergent was removed through the addition of Bio-Beads and exchanging them every 8 h for several days. Reconstitutions were confirmed by negative stain electron microscopy and HS-AFM imaging.

### HS-AFM imaging

In total, 1.5 µl of the TRPV2 reconstituted vesicles were deposited on a 1.5-mm^2^ freshly cleaved mica surface, which was glued with epoxy to the quartz sample stage. After 20–30 min incubation, the sample was gently rinsed with imaging buffer (20 mM Hepes, pH 8.0, 150 mM NaCl) and mounted in the HS-AFM fluid cell. All images in this study were taken using a HS-AFM (Research Institute of Biomolecule Metrology Co.) operated in amplitude modulation mode using optimized scan and feedback parameters and lab-built amplitude detectors and free amplitude stabilizers^41,42^. Short (8 µm) cantilevers (USC-F1.2-k0.15, NanoWorld) with nominal spring constant of 0.15 N/m, resonance frequency of 0.6 MHz, and a quality factor of ∼1.5 in liquid were used. AFM probes were sharpened using oxygen plasma etching to obtain better resolution.

### Calculation of correlation average and localization AFM (LAFM) maps

HS-AFM movies were drift-corrected using lab-made plugins in ImageJ. The correlation average map was calculated using home-made plugins in ImageJ in the following way: (1) A single TRPV2 molecule was chosen from a data set. (2) This molecule was four-fold symmetrized and used as reference-1 to search for molecules in the respective HS-AFM movie in a first round of cross-correlation searches. (3) From this first round of cross-correlation searches an average was calculated that served as reference-2. (4) This reference was four-fold symmetrized and used for a second round of cross-correlation searches. (5) All found particles were extracted, merged in a stack and used to calculating an average map, where the value of each pixel x_*n*_, y_*n*_ is the average value of all pixels *x*_*n*_*, y*_*n*_ in all molecules (Fig. 2c). To calculate a LAFM map, the extracted particles were expanded to 0.5 Å pixel^−1^ pixel-sampling using bicubic interpolation and further laterally and rotationally aligned using lab-made plugins in ImageJ. Then we applied the LAFM algorithm that detects local maxima, extracts their height, and merges height and peaking probabilities to produce a LAFM map^20^ (Fig. 2d).

### IDR prediction based on sequence

We used several web-based computational tools to predict the disorder propensity in the TRPV2 sequence. Although the different tools use different methods to extract disorder propensity, they rely on characteristics such as hydropathy, charge, and sequence complexity. In this work we used (1) predictor of naturally disordered regions (PONDR), a feedforward neural network, for which we used scores from three predictors VLXT, VL3, and VLS2. (2) IUPred—a method based on estimating pairwise interaction energies from amino acid composition. (iii) PONDR-FIT—a meta predictor that combines different predictors such as PONDR-VLXT, PONDR-VSL2, PONDR-VL3, and IUPred. (iv) PRODOS. All these tools provided output disorder score that ranged between 0 and 1. We averaged all predictions and calculated the mean and standard deviation value for each residue (Fig. 2j).

### Hydrophobic score based on sequence

We used the ExPasy protScale software to calculate the hydrophobic score of the full-length TRPV2 sequence. An average score and standard deviation along the sequence was calculated from several hydropathy scales, Kyte & Doolittle, Abraham & Leo, Roseman, Wolfeden, Black, Fauchere, and Rao & Argos (Fig. 2k).

### Identification of unresolved stretches in TRPV2 structures

First, all residues in the seven currently available structures of *Rattus norvegicus* TRPV2 structures were aligned. Second, unresolved residues were assigned to a score of 0, while resolved residues were assigned a score of 1. Third, scores were summed for each residue in all seven structures. Then, scores of all structures were plotted by stacking them to each other with the sum overlaid (Fig. 2l).

### Flexible-meccano modeling of terminal IDRs

The flexible-meccano algorithm builds multiple, different copies of the same polypeptide chain by randomly sampling the potential wells of amino acid-specific backbone dihedral angles {φ/ψ}. The population-weighted potentials were derived from a compilation of non-secondary structural elements of high-resolution X-ray structures^35^ that have been extensively validated against atomic resolution NMR data, and shown to represent the ensemble of disordered states sampled by IDRs in solution^43^. The selected {φ/ψ} pairs are used to sequentially connect peptide planes and thus build peptide chains. To avoid steric clashes (volume exclusion) amino acid are modeled as residue specific hard-spheres that serve as repulsive forces. No attractive forces are used. Twenty-three potential energy wells are sampled: one for each of the 20 amino acids, and specific potentials for the particular backbone conformational propensities of residues that precede prolines, prolines that precede prolines, and glycines that precede prolines. Thus, 20,000 structures of the N- and C-terminal IDRs were calculated associated to the channel cryo-EM structure to make sure that IDRs could only explore the conformational space outside the protein core (Fig. 2g). From the 20,000 IDR predictions, a 4-fold symmetrized 3D density map was calculated reflecting the reach and distribution of the IDR termini as density clouds (Supplementary Fig. 2). The flexible-meccano predictions were performed in the absence of a membrane.

### Principal component analysis (PCA)

Each particle of a movie M (a 3-dimensional x*y*t array) was reshaped into a 2-dimensional array X (xy*t, where each column contains a movie frame reshaped into a 1-dimentional array, and each row corresponds to a different frame t in a movie). Principal component coefficients (contained in a new 2-dimentional array C, size t*t) were calculated using the Matlab pca function that uses a singular value decomposition (SVD) algorithm. The particle movie in the new space Y (size xy*t) was calculated by multiplying X with C. Y contains principal component images as 1-dimentional vectors sorted in descending order of the principal component variances (Fig. 2h).

### TRPV2-TRPV2 interaction analysis

Protein-protein interactions were analyzed in a following way: (1) HS-AFM movie frames were cropped around regions containing only 2 or 3 interacting TRPV2 tetramers. (2) The cropped frames were further aligned, filtered to remove noise, and binarized in ImageJ. (3) In the binarized frames, connected objects could be computationally identified using the analyze particles routine in ImageJ. (4) When the channels were connected (detected as one continuous particle), a score of 1 was assigned, in contrast, when the proteins were disconnected (detected as two individual areas in ImageJ), a score of 0 was assigned. (5) From this two-state assignment (connected or not), connection dwell-times were calculated, from which (6) a survival plot was calculated and fitted with an exponential function to deduce the time constant (Fig. 3b).

### TRPV2-TRPV2 IDR angular analysis

We extracted protein-protein interacting pairs from drift-corrected movies and further aligned the pairs. Using ImageJ, the centers of each protomer were detected in each molecule in all movie frames. Then, the center of each tetramer was calculated from the geometric mean of the centers of the four protomers. Next, we calculated distances lines from the tetramer centers of the molecules to corresponding protomers that participated in the interactions. Finally, we calculated the angles from the positions of the tetramer centers and the protomers. All angle detection from both interacting molecules were pooled and to generate a polar coordinate histogram plot (Fig. 3c).

### Statistics and reproducibility

The data presented in the paper comes from at least three different independent biological replicates, i.e., TRPV2 purifications, and reconstitution matrices (varying LPRs). The HS-AFM images are from at least five best different imaging sessions, which gave the highest resolution data representative for at least 50 HS-AFM imaging sessions. The HS-AFM images have been acquired using between 5 and 10 different HS-AFM tips.

### Reporting summary

Further information on research design is available in the Nature Portfolio Reporting Summary linked to this article.

### Supplementary information

Supplementary Figures

Description of Additional Supplementary Data

Video 1

Video 2

Video 3

Video 4

Video 5

Video 6

Reporting Summary

### Source data

Source Data File

## Data Availability

Source Data of all graph figure panels are available in a source data file Supplementary Data 1. All other data needed to evaluate the conclusions in the paper are presented in the paper and the other Supplementary Materials. Additional data and materials related to this paper may be requested from the authors. Source data are provided with this paper.

## Code availability

All data analysis code used in this paper, calling for built-in functions in ImageJ and Matlab as detailed in the “Methods” section, may be requested from the authors.
