# Supplementary material for: Intrinsically disordered regions in TRPV2 mediate protein-protein interactions
Source: Commun Biol. 2023 Sep 22;6:966. doi: 10.1038/s42003-023-05343-7 (PMC10516966; doi:10.1038/s42003-023-05343-7)
Supplement: Supplementary file 1 — Supplementary Figures [file 42003_2023_5343_MOESM1_ESM.pdf]

SUPPLEMENTARY FIGURES

Supplementary Figure S1

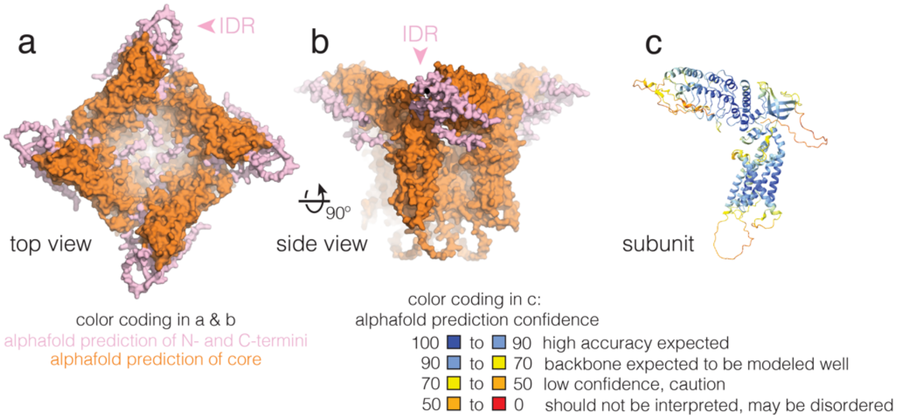

Supplementary Figure S1 | Detailed view on the N-terminal AlphaFold IDR structure prediction. (a) Top view and **(b)** side view, of the TRPV2 channel with the channel core structure in orange and the IDRs in pink (surface representation). AlphaFold predicts, likely due to the resolution of residues ~30 to ~45 in some structures adhering to the membrane facing side of the ARDs, the N-terminal IDR to loop at the periphery of the channel (arrowhead). (c) TRPV2 protomer colored by structure prediction confidence: The IDRs are predicted with 'low confidence' or 'may be disordered'.

Supplementary Figure S2

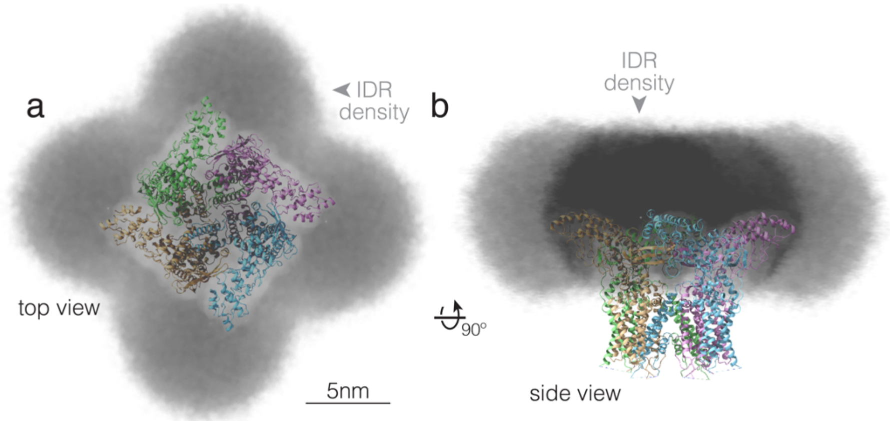

Supplementary Figure S2 | Density map calculated from flexible-meccano IDR structure prediction. (a) Top view and **(b)** side view, of the TRPV2 channel with the channel core structure shown in ribbon representation and the four subunits in different colors. Grey clouds: 3D density calculated from the Flexible-meccano predicted IDRs (see main text Figure 2g). The density extends with high probability beyond >5nm in good agreement with the inter-molecular connections between TRPV2 channels (see main text Figure 3).
