# Supplementary material for: Intrinsically disordered regions in TRPV2 mediate protein-protein interactions
Source: Commun Biol. 2023 Sep 22;6:966. doi: 10.1038/s42003-023-05343-7 (PMC10516966; doi:10.1038/s42003-023-05343-7)
Supplement: Supplementary file 2 — Description of Additional Supplementary Data [file 42003_2023_5343_MOESM2_ESM.pdf]

DESCRIPTION OF ADDITIONAL SUPPLEMENTARY DATA

Supplementary Movies Captions:

Supplementary Movie 1: HS-AFM movie of TRPV2 in lipid bilayers in 20 mM Hepes, pH 8.0, 150 mM NaCl buffer, recorded at 1 frame/s. Right panel: Time average over 3 seconds. Scale bar: 20 nm.

Supplementary Movie 2: HS-AFM movie of TRPV2 in lipid bilayers in 20 mM Hepes, pH 8.0, 150 mM NaCl buffer, recorded at 1 frame/s. Right panel: Time average over 3 seconds. Scale bar: 10 nm.

Supplementary Movie 3: HS-AFM movie of TRPV2 in lipid bilayers in 20 mM Hepes, pH 8.0, 150 mM NaCl buffer, recorded at 1 frame/s. Right panel: Time average over 3 seconds. Scale bar: 10 nm.

Supplementary Movie 4: HS-AFM movie of TRPV2 in lipid bilayers in 20 mM Hepes pH 8.0, 150 mM NaCl buffer, recorded at 1 frame/s. Right panel: Time average over 3 seconds. Scale bar: 10 nm.

Supplementary Movie 5: HS-AFM movie of TRPV2 in lipid bilayers in 20 mM Hepes pH 8.0, 150 mM NaCl buffer, recorded at 1 frame/s. Right panel: Time average over 3 seconds. Scale bar: 10 nm.

Supplementary Movie 6: HS-AFM movie of TRPV2 in lipid bilayers in 20 mM Hepes pH 8.0, 150 mM NaCl buffer, recorded at 6 frames/s. Right panel: Time average over 0.5 second. Scale bar: 10 nm.

Source Data:

A Source Data XCEL file is provided tabulating all graphed figure panels 1c, 2j, 2k, 2l, 3a, 3b, and 3c.
