# Supplementary material for: Intrinsically disordered regions in TRPV2 mediate protein-protein interactions
Source: Commun Biol. 2023 Sep 22;6:966. doi: 10.1038/s42003-023-05343-7 (PMC10516966; doi:10.1038/s42003-023-05343-7)
Supplement: Supplementary file 9 — Reporting Summary [file 42003_2023_5343_MOESM9_ESM.pdf]

nature portfolio

Corresponding author(s): Simon SCHEURING

Last updated by author(s): Aug 22, 2023

Reporting Summary

Nature Portfolio wishes to improve the reproducibility of the work that we publish. This form provides structure for consistency and transparency in reporting. For further information on Nature Portfolio policies, see our Editorial Policies and the Editorial Policy Checklist.

Statistics

For all statistical analyses, confirm that the following items are present in the figure legend, table legend, main text, or Methods section.

n/a	Confirmed	
☐	☒	The exact sample size (*n*) for each experimental group/condition, given as a discrete number and unit of measurement
☒	☐	A statement on whether measurements were taken from distinct samples or whether the same sample was measured repeatedly
☒	☐	The statistical test(s) used AND whether they are one- or two-sided<br>*Only common tests should be described solely by name; describe more complex techniques in the Methods section.*
☒	☐	A description of all covariates tested
☒	☐	A description of any assumptions or corrections, such as tests of normality and adjustment for multiple comparisons
☐	☒	A full description of the statistical parameters including central tendency (e.g. means) or other basic estimates (e.g. regression coefficient) AND variation (e.g. standard deviation) or associated estimates of uncertainty (e.g. confidence intervals)
☒	☐	For null hypothesis testing, the test statistic (e.g. *F*, *t*, *r*) with confidence intervals, effect sizes, degrees of freedom and *P* value noted<br>*Give P values as exact values whenever suitable.*
☒	☐	For Bayesian analysis, information on the choice of priors and Markov chain Monte Carlo settings
☒	☐	For hierarchical and complex designs, identification of the appropriate level for tests and full reporting of outcomes
☒	☐	Estimates of effect sizes (e.g. Cohen's *d*, Pearson's *r*), indicating how they were calculated

Our web collection on statistics for biologists contains articles on many of the points above.

Software and code

Policy information about availability of computer code

Data collection	Igor Pro 7 on HS-AFM SS-NEX (RIBM)
Data analysis	HS-AFM image analysis: Igor Pro 7, MATLAB R2019b, ImageJ 1.52 / IDR prediction of the TRPV2 sequence: We used (i) predictor of naturally disordered regions (PONDR), a feedforward neural network, for which we used scores from three predictors VLXT, VL3, and VLS2. (ii) IUPred- a method based on estimating pairwise interaction energies from amino acid composition. (iii) PONDR-FIT-a meta predictor that combines different predictors such as PONDR-VLXT, PONDR-VSL2, PONDR-VL3, and IUPred. (iv) PRODOS / Hydrophobic score of the TRPV2 sequence: We used ExPasy protScale software to calculate the hydrophobic score from several hydropathy scales, Kyte & Doolittle, Abraham & Leo, Roseman, Wolfeden, Black, Fauchere, and Rao & Argos / The full length TRPV2 structure prediction including the terminal IDRs was downloaded from the AlphaFold website (https://alphafold.ebi.ac.uk) / The ensemble structure prediction of the IDRs was computed using flexible-meccano (https://www.ibs.fr/en/communication-outreach/scientific-output/software/flexible-meccano-en?lang=en)

For manuscripts utilizing custom algorithms or software that are central to the research but not yet described in published literature, software must be made available to editors and reviewers. We strongly encourage code deposition in a community repository (e.g. GitHub). See the Nature Portfolio guidelines for submitting code & software for further information.

Data

Policy information about availability of data

All manuscripts must include a data availability statement. This statement should provide the following information, where applicable:

- Accession codes, unique identifiers, or web links for publicly available datasets
- A description of any restrictions on data availability
- For clinical datasets or third party data, please ensure that the statement adheres to our policy

All data needed to evaluate the conclusions in the paper are present in the paper and the Supplementary Materials. Additional data related to this paper may be requested from the authors.

Human research participants

Policy information about studies involving human research participants and Sex and Gender in Research.

Reporting on sex and gender	N/A
Population characteristics	N/A
Recruitment	N/A
Ethics oversight	N/A

Note that full information on the approval of the study protocol must also be provided in the manuscript.

Field-specific reporting

Please select the one below that is the best fit for your research. If you are not sure, read the appropriate sections before making your selection.

☒ Life sciences ☐ Behavioural & social sciences ☐ Ecological, evolutionary & environmental sciences

For a reference copy of the document with all sections, see nature.com/documents/nr-reporting-summary-flat.pdf

Life sciences study design

All studies must disclose on these points even when the disclosure is negative.

Sample size	No method was used to predefine the sample size. The height of TRPV was defined from >5 million pixels from supplementary Movie 1. The HS-AFM average map (n=532 particles) and LAFM map (n=532 particles, 80,000 localization detections) were calculated from a larger number of particles than previously shown to saturate the signal and being statistically significant. The distributions of lifetime of IDR interactions and IDR orientations were pooled from >500 IDR observations, a typical number for single molecule studies.
Data exclusions	No data was excluded
Replication	The data presented represent the best data from >20 conclusive and agreeing experiments from >5 biological replicates (protein samples and reconstitutions)
Randomization	No randomization was needed and performed for such a bio-molecular study.
Blinding	No blinding was needed and performed for such a bio-molecular study.

Reporting for specific materials, systems and methods

We require information from authors about some types of materials, experimental systems and methods used in many studies. Here, indicate whether each material, system or method listed is relevant to your study. If you are not sure if a list item applies to your research, read the appropriate section before selecting a response.

Materials & experimental systems

n/a	Involved in the study
☒	☐ Antibodies
☒	☐ Eukaryotic cell lines
☒	☐ Palaeontology and archaeology
☒	☐ Animals and other organisms
☒	☐ Clinical data
☒	☐ Dual use research of concern

Methods

n/a	Involved in the study
☒	☐ ChIP-seq
☒	☐ Flow cytometry
☒	☐ MRI-based neuroimaging
